# Optical, Structural, and Synchrotron X-ray Absorption Studies for GaN Thin Films Grown on Si by Molecular Beam Epitaxy

**DOI:** 10.3390/ma17122921

**Published:** 2024-06-14

**Authors:** Zhe Chuan Feng, Jiamin Liu, Deng Xie, Manika Tun Nafisa, Chuanwei Zhang, Lingyu Wan, Beibei Jiang, Hao-Hsiung Lin, Zhi-Ren Qiu, Weijie Lu, Benjamin Klein, Ian T. Ferguson, Shiyuan Liu

**Affiliations:** 1 State Key Laboratory of Intelligent Manufacturing Equipment and Technology, Huazhong University of Science and Technology, Wuhan 430074, China; zcfeng@ntu.edu.tw (Z.C.F.); chuanweizhang@hust.edu.cn (C.Z.); shyliu@hust.edu.cn (S.L.); 2 Southern Polytechnic College of Engineering and Engineering Technology, Kennesaw University, Marietta, GA 30060, USA; mnafisa@students.kennesaw.edu (M.T.N.); bjiang1@kennesaw.edu (B.J.); bklein8@kennesaw.edu (B.K.); ianf@kennesaw.edu (I.T.F.); 3 Science Exploring Lab, Arbour Glenn Drive, Lawrenceville, GA 30043, USA; 4 School of Electronic & Electrical Engineering and Physics, Fujian University of Technology, Fuzhou 350118, China; 5 Center on Nano-Energy Research, Laboratory of Optoelectronic Materials & Detection Technology, Guangxi Key Laboratory for the Relativistic Astrophysics, School of Physical Science & Technology, Guangxi University, Nanning 530004, China; lyw2017@gxu.edu.cn; 6 Department of Electrical Engineering, Graduate Institute of Photonics and Optoelectronics, National Taiwan University, Taipei 10617, Taiwan; hhlin@ntu.edu.tw; 7 State Key Laboratory of Optoelectronic Materials and Technologies, School of Physics, Sun Yat-sen University, Guangzhou 510275, China; stsqzr@mail.sysu.edu.cn; 8 Hexagonal Scientific Lab, LLC, Dayton, OH 45459, USA; wlu@hsl-mat.com

**Keywords:** gallium nitride (GaN), molecular beam epitaxy (MBE), high-resolution X-ray diffraction (HR-XRD), Nomarski microscopy (NM), Raman scattering, photoluminescence (PL), spectroscopic ellipsometry (SE), Urbach’s binding energy, synchrotron radiation (SR), near-edge X-ray absorption fine structure (NEXAFS)

## Abstract

GaN on Si plays an important role in the integration and promotion of GaN-based wide-gap materials with Si-based integrated circuits (IC) technology. A series of GaN film materials were grown on Si (111) substrate using a unique plasma assistant molecular beam epitaxy (PA-MBE) technology and investigated using multiple characterization techniques of Nomarski microscopy (NM), high-resolution X-ray diffraction (HR-XRD), variable angular spectroscopic ellipsometry (VASE), Raman scattering, photoluminescence (PL), and synchrotron radiation (SR) near-edge X-ray absorption fine structure (NEXAFS) spectroscopy. NM confirmed crack-free wurtzite (w-) GaN thin films in a large range of 180–1500 nm. XRD identified the w- single crystalline structure for these GaN films with the orientation along the c-axis in the normal growth direction. An optimized 700 °C growth temperature, plus other corresponding parameters, was obtained for the PA-MBE growth of GaN on Si, exhibiting strong PL emission, narrow/strong Raman phonon modes, XRD w-GaN peaks, and high crystalline perfection. VASE studies identified this set of MBE-grown GaN/Si as having very low Urbach energy of about 18 meV. UV (325 nm)-excited Raman spectra of GaN/Si samples exhibited the GaN E_2_(low) and E_2_(high) phonon modes clearly without Raman features from the Si substrate, overcoming the difficulties from visible (532 nm) Raman measurements with strong Si Raman features overwhelming the GaN signals. The combined UV excitation Raman–PL spectra revealed multiple LO phonons spread over the GaN fundamental band edge emission PL band due to the outgoing resonance effect. Calculation of the UV Raman spectra determined the carrier concentrations with excellent values. Angular-dependent NEXAFS on Ga K-edge revealed the significant anisotropy of the conduction band of w-GaN and identified the NEXAFS resonances corresponding to different final states in the hexagonal GaN films on Si. Comparative GaN material properties are investigated in depth.

## 1. Introduction

The wide direct bandgap and excellent thermal stability properties of the GaN-based materials have promoted their commercial and potential application to light-emitting diodes (LEDs), laser diodes (LDs), photodetectors (PDs), high-power and high-frequency electronic devices [1,2,3,4], as well as GaN-on-Si high-electron mobility transistor (HEMT) [5]. With respect to the growth of these III-nitride materials and structures, sapphire and silicon carbide (SiC) are commonly used as substrates, and metalorganic chemical vapor deposition (MOCVD) technology has been extensively implemented for the industry mass production of III-nitride materials [1,2]. However, their use is limited by the insulating property of sapphire and the expensive cost of SiC. Therefore, other substrate materials have been explored for replacement. Silicon (Si) is a good candidate offering more advantages in comparison with SiC and sapphire, and is associated with crystalline perfection, a large area size, a low manufacturing cost, excellent electrical and thermal conductivity, being very suitable as a substrate for the growth of III-N materials and structures. Therefore, the growth of GaN-based materials and structures on Si has attracted considerable attention during this century [5,6,7,8,9,10,11,12,13,14,15,16]. Currently, the investigation into the growth and properties of GaN–Si materials and devices represents a hot topic in research and development (R&D) [17,18,19,20,21,22,23,24]. However, between GaN and Si, there exists a large lattice mismatch (17%) and a big difference in expansion coefficients [18,19], causing more difficulties in relation to the growth of high-quality GaN films and structures on Si relative to the growth of GaN films on sapphire. To overcome these difficulties, various buffer layers and composite structures have been adopted [6,7,8,10,12,13,14,18,19,22]. In addition to the MOCVD growth of III-nitrides on Si [12,13,19,22], molecular beam epitaxy (MBE) has been explored for the growth of epitaxial GaN on Si [6,7,8,9,23]. Among the above, buffer structures that have been employed thus far include AlN buffer [6,8,12,13,14,19,22], AlN/GaN superlattice [10], AlGaN buffer [18], and so on. Also, the GaN layer quality and characteristics, including stress and cracking, probed by various characterization techniques, have been shown to influence the fabrication and performance of devices on Si, such as HEMT [10,13], LEDs [12,20], power devices [16,23], photonic devices [24], and so on. Therefore, it is very important to obtain multi-technological and comprehensive characterizations of GaN layers and of the structures grown on Si.

In the present work, a unique plasma assistant (PA) MBE using pulsed source injection (PSI) was employed to grow a GaN layer on Si with a thin AlN buffer [6,8]. This technique provides a suitable relaxation time for the two-dimensional growth to avoid the problem of the 3D nucleation of GaN grown on Si substrate. By way of the PAMBE PSI process, a series of thin GaN films were successfully deposited on Si (111) substrates. These thin GaN films were characterized and studied using multiple techniques such as Nomarski microscopy (NM), high resolution X-ray diffraction (HR-XRD), spectroscopic ellipsometry (SE), Raman scattering (RS), photoluminescence (PL), and synchrotron radiation (SR) near-edge X-ray absorption fine structure (NEXAFS) spectroscopy. A comparative investigation using visible Raman, ultraviolet (UV) Raman–PL, and NEXAFS was performed to explore further material properties in depth and to serve for further device manufacturers.

## 2. Materials and Methods

The experimental GaN thin films on Si substrates were prepared using the plasma assistant molecular epitaxy (PA-MBE) and the pulsed source injection (PSI) technique. W. Tong et al. [6] described this MBE technique and the growth procedure of GaN on Si (111) in detail. Silicon (111) substrates were used to deposit first an AlN buffer layer at growth temperatures between 650 °C and 750 °C and with an AlN buffer layer thickness less than 10 nm. Subsequently, the GaN growth was promoted continually with growth temperatures between 550 °C and 700 °C. An N flow rate of 0.6 sccm was used for the GaN growth across all samples. The Ga flux used was between 1.9 × 10^−7^ torr and 2.9 × 10^−7^ torr. The two-dimensional growth of GaN was achieved through monitoring with in situ reflection high-energy electron diffraction (RHEED).

A series of seven MBE-grown GaN/Si samples were used in the present study, with their information listed in Table 1, including some characterization results as described in the next sections. In earlier reports [6,8], some primary results have been presented for some pieces of samples with very limited measurements and analyses. Here, a comprehensive investigation into the structural, surface, and optical properties of MBE-grown GaN on Si was performed penetrative by employing multiple technologies, including Nomarski microscope (NM), high resolution X-ray diffraction (HRXRD), spectroscopic ellipsometry (SE), visible and ultraviolet Raman scattering, photoluminescence (PL), synchrotron radiation (SR), and near-edge X-ray absorption fine structure (NEXAFS). An Olympus Nomarski microscope (NM) was used for the assessment of surface morphology. High resolution X-ray diffraction (HR-XRD) scans were measured by using a Phillips MRD five-crystal system. Visible Raman scattering (RS) spectra were obtained from a Renishaw micro-Raman system, under an excitation of 532 nm. UV excitation (325 nm from a He–Cd laser) combined with Raman–photoluminescence (PL) measurements were performed using a Jobin Yvon-Horiba T64000 three grating high-resolution system (Horiba, Kyoto, Japan) and a Renishaw UV micro-Raman–PL system. Detailed data and analyses from these measurements will be given in the next sections.

A Filmetrics F20UV thin film measurement instrument was initially used to measure the UV visible optical reflectance (OR) in 250–850 nm and to determine the film thickness for the MBE growth control and adjustment of GaN/Si [6]. Further careful measurements and simulations were conducted using the variable angle spectroscopic ellipsometry (VASE) technique, using a dual rotating compensator Mueller matrix ellipsometer from Wuhan Eoptics Technology Co. Ltd., Wuhan, China. The measured RT SE data were simulated (detailed description in Section 3.3) and the seven GaN film thicknesses are listed in Table 1.

Angle-dependent Ga *K*-edge NEXAFS measurements were performed at the National Synchrotron Radiation Research Center (NSRRC), at beamline 17C and taken in the fluorescence mode. The XANES spectra were acquired with beam incident angles from 15° to 90° between the direction of the incident photon and film surface normal orientation.

## 3. Results

### 3.1. Surphase Morphology and Nomarski Microscopy

Having monitored MBE growth previously, the reflection high-energy electron diffraction (RHEED) examinations were performed on this set of GaN on Si samples. The in situ RHEED displayed sharp and clear streaky patterns, indicative of two-dimensional layer-by-layer growth and good surface morphology [6]. RHEED can be used to monitor the MBE growth. The oscillations of the specular beam intensity indicate a layer-by-layer growth which allows one to precisely measure the deposition rate [7]. The RHEED intensity oscillations were monitoring the interface roughness and demonstrated the existence of flat surfaces [9]. The GaN film surface morphology was studied by atomic force microscopy [11,12,13,14,22]. Our MBE-grown GaN films on Si were also examined via AFM and AFM studies have shown that an almost atomically smooth surface is obtained with an area root mean square (RMS) roughness of less than 0.2 nm [6].

**Figure 1:**
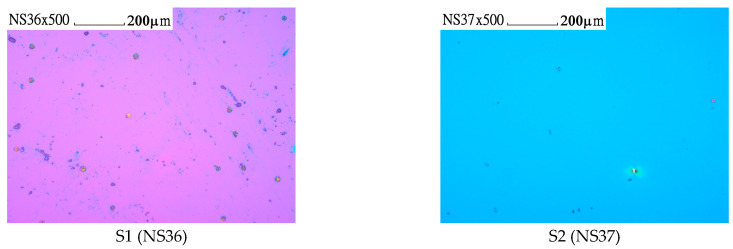

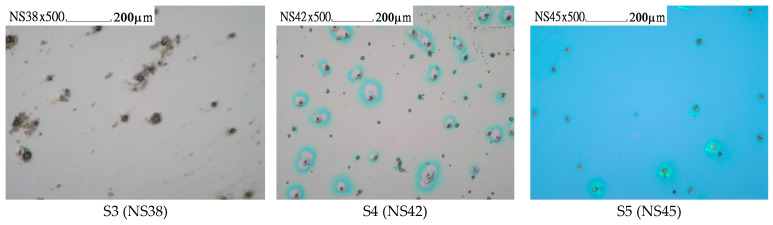
Nomarski microscopy patterns of five GaN films grown on Si by MBE. All photos were taken with a ×500 magnification and a ruler length of 200 μm.

Here, the Olymbus Nomarski (NM) microscope was employed for further assessments of the surface morphology to confirm the GaN film formed without cracks. Figure 1 shows NM photos, taken with a ×500 magnification and with a ruler length of 200 μm on five GaN/Si samples. There were no cracks observed within the samples. The cracking is an important technological problem in the growth of III-nitride (III-N) layers on Si [23], causing troubles for the fabrication of III-N HEMT [10,13] and LED [12] devices on Si. It has previously been reported that cracks may appear in the GaN films grown on Si by metal-organic chemical vapor deposition (MOCVD) [25,26]. In [26], we reported the use of NM for surface morphology examination which helped for the cracking control of MOCVD GaN/Si successfully. M.-H. Kim et al. [27] pointed out that a big difference in thermal expansion coefficients between GaN and Si could cause a large tensile stress in the GaN layer, leading to cracks in GaN formed during the cooling stage. T. Malin et al. [23] demonstrated that the lattice mismatch between III-nitrides and Si (111) (~19%) and their thermal expansion coefficient difference (~33%) can result in the formation of high-density defects of various types, including cracks, in the III-N epitaxial layer on Si (111) substrates during post-growth cooling. Various buffer structures have been employed for the MOCVD growth of crack-free hexagonal GaN layers on Si, such as an AlN buffer [14,19], AlGaN buffer [18,22], AlN/GaN superlattice buffer [10], step-graded AlGaN buffer [12], AlN/AlGaN buffer [28], and more. Our present work demonstrates the success of crack-free GaN films grown on Si from MBE. It is worthy to note that these crack-free GaN films have a wide range of thicknesses, from less than 200 nm up to 1.5 μm.

### 3.2. Structural Characterization by High-Resolution X-ray Diffraction

**Figure 2:**
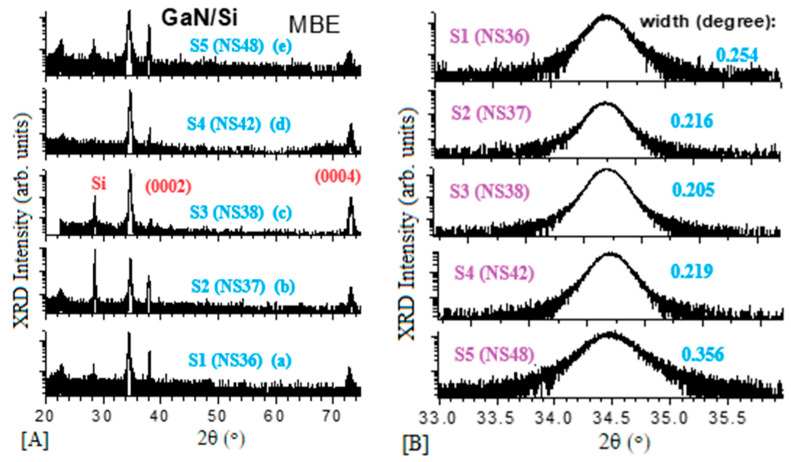
High resolution XRD measurements of five MBE-grown GaN/Si, (**A**) scans between 20–75 degrees of 2-theta and (**B**) w-GaN (0002) mode and their widths.

High-resolution X-ray diffraction (HR-XRD) is a useful technique to characterize crystalline perfection. The GaN epitaxial layer can be characterized by HR-XRD 2θ-ω scans for the surface orientation. Figure 2A exhibits HR-XRD wide (20–75°) 2θ−ω scans for five MBE GaN/Si samples. All show mainly the (0002) and (0004) peaks from wurtzite (w-) GaN films and peaks from the Si substrate. There are no peaks related to other orientated GaN peaks to be observed. It can be determined that these five GaN samples grown on the Si substrate possessed all the (0001) orientation, i.e., along the c-axis of the wurtzite GaN structure. Figure 2B presents narrow scans of the GaN (0002) peaks, with the values of full width at half maximum (FWHW), obtained by gaussian fitting for all samples, which are indicated in the figure and listed in Table 1. As it can be seen, the sample N3 (NS38, 1.5 μm thick) possessed the strongest (0002) and (0004) w-GaN peaks and the narrowest (0002) w-GaN peak. The N4 (NS42, 547 nm) sample had the second strongest (0002) and (0004) GaN peaks, while the N5 (NS45) sample exhibited the weakest peaks.

To analyze the structural features of five GaN/Si samples and compare their crystal characteristics, further quantitative calculations were performed. First, the average crystallite size of five GaN films can be evaluated using the Debye–Sheller formula [15,29]:

(1) $$D=\frac{k\lambda }{\beta cos\theta }$$

where *D* is the crystallite size, *β* is the full width at half maximum of the (0002) XRD peak, *k* is the Scherrer constant that is 0.9, *θ* is the diffraction angle, and *λ* is the X-ray diffraction wavelength which is 1.5406 Å.

In addition, the microscopic strain (*ε*) can be calculated using the following expression [29]:

(2) $$\varepsilon =\frac{\beta cos\theta }{4}$$

with *β* and *θ* defined as above.

The dislocation density of GaN can be calculated through the following formula [15]:

δ = 1/D^2^ (3)

By way of these calculations, the crystallite size, micro-strain, and screw dislocation density values of five GaN films were obtained and they are listed in Table 2. It was found that sample S3 (NS38) possessed the narrowest FWHM, the widest crystallite size, the lowest lattice strain, and the lowest dislocation density, while the sample S5 (NS45) exhibited the worst.

### 3.3. Spectroscopic Ellipsometry Measurements and Analysis of GaN Films on Si

From spectroscopic ellipsometry (SE) experiments, the polarization states psi (Ψ) and delta (Δ) between the incidence and reflection of light on a sample and associated variations were measured against the wavelength. The layer thickness, surface roughness, and optical constants of the GaN epitaxial layer were derived from SE data. SE spectra of GaN samples were fitted via J A Woollam CompleteEASE software (https://www.jawoollam.com/ellipsometry-software/completeease) by establishing a multi-layer physical model. Variable angle spectroscopic ellipsometry (VASE) measurements employ multiple incident angles and lead to the simulation that fits most accurately. For our experimental samples of GaN/Si with an AlN buffer, the optical constants were taken for the Si substrate with Si_JAW3, for the AlN buffer with AlN_g, and for the GaN layer with GaN_g, all from J A Woollam software library.

**Figure 3:**
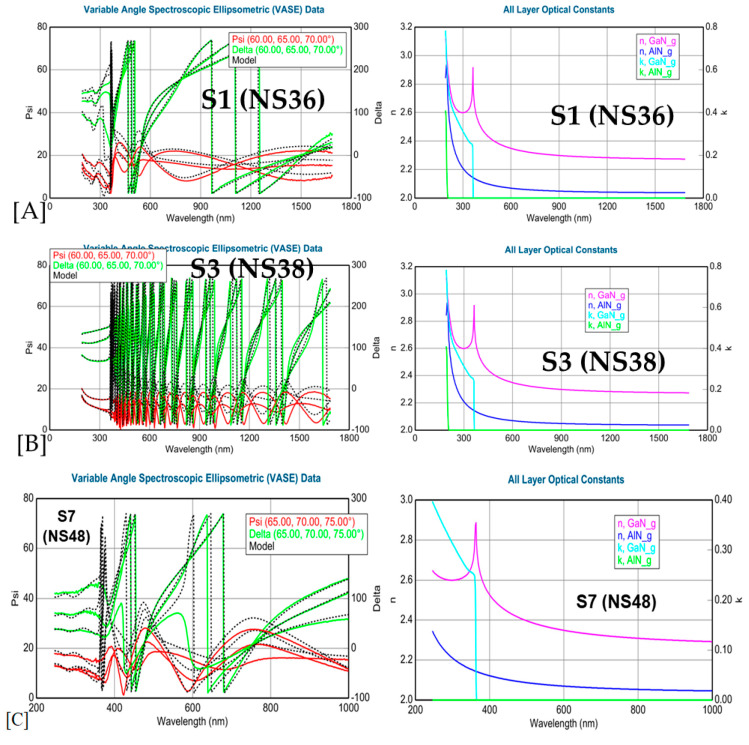

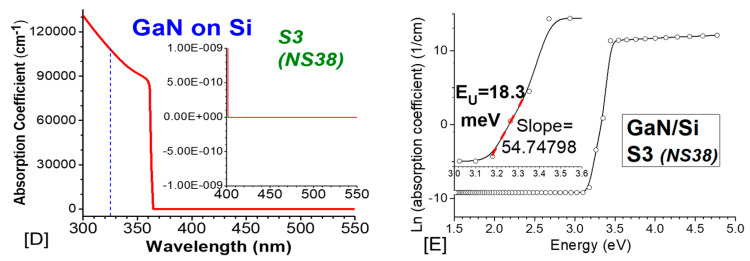
SE spectra and fits: (**A**,**B**) experimental and fitted psi (Ψ) and delta (Δ) spectra vs. wavelength (193–1700 nm) under three angle incidences of 65°, 70°, and 75° at RT for samples S1 and S3, (**C**) psi (Ψ)/delta (Δ) and n and k spectra in 250–1000 nm for sample S7, (**D**) absorption coefficient α in 300–550 nm for sample S3, and (**E**) ln(α) in 1.5–5.0 eV with insert on the Urbach energy E_U_ for sample S3.

Figure 3 presents variable angle (VA) SE spectra and fitting results: (A,B) experimental and fitting curves of psi (Ψ) and delta (Δ) vs. nm (193–1700 nm) under three angle incidences of 65°, 70°, and 75° at RT for the samples S1 and S3, (C) psi (Ψ) and delta (Δ) vs. nm spectra, n and k vs. nm spectra in 250–1000 nm for sample S7, (D) absorption coefficient α~λ in 300–550 nm, and (E) ln(α)~eV in 1.5–5.0 eV plus insert on the Urbach energy E_U_ for sample S3.

From the SE spectral simulation results, GaN film thicknesses and surface roughness (including the AlN buffer layer thicknesses) were obtained and they are listed in Table 3. From the deduced GaN n~λ curves, the sharp peaks indicated indeed the bandgap of GaN, which matched well the middle points of the steep drops from the GaN k~λ curves. These obtained E_g_(GaN) values in units of both wavelength (nm) and energy (eV) are listed in Table 3. The seven GaN samples had a bandgap energy ranging from 3.415 eV to 3.419 eV with a difference within 0.004 eV. Similarly, the AlN buffer n~λ curves showed sharp peaks with the E_g_(AlN) in the energy range of 6.274 eV to 6.284 eV. For sample S7, the experimental SE spectra were measured in a shorter range of 250–1000 nm, but these were quite good for the determination and analysis of the GaN energy gap and film information, although not for the AlN buffer gap value. From Figure 3D, the SE deduced α~λ relation is displayed for sample S3 in the near E_g_(GaN) range of 300–550 nm. Indeed, all other six samples had similar α~λ curves to S3 because of their Eg values being ± 0.004 eV, i.e., with ~0.1% error bars.

Usually, plots of (*αhv*)^2^ versus photon energy are applied to obtain the bandgaps for GaN [29,30] and other materials. The relationship between the absorption coefficient (α) and photon energy (eV) is described by the equation [31]:

(4) $$\alpha hv=C{\left(hv-E_{g}\right)}^{1/2}$$

where hv is the photon energy, $E_{g}$ is the band gap of the semiconductor, and C is a constant. To extrapolate the linear part of the curve of (*αhν*)^2^ vs. (*hν* − *E_g_*) to the x-axis, i.e., the so-called Tauc plot [31], the GaN bandgap energy (*E_g_*) was obtained. We tried this method on our GaN/Si samples, as our team did for GaN/sapphire [29] and obtained *E_g_* values almost exactly identical to what we obtained from the peaks of n~λ curves, as listed in Table 3. Also, our seven GaN films with large film thickness variation between 160–1500 nm had their *E_g_* values with only ~0.1% differences, indicating the excellent GaN film quality grown on Si by MBE.

From Figure 3E, an exponential absorption band tail below the band-edge of the sample can be observed. Y. Liu et al. [32] predicted that it may be caused by the structural disorder accompanying electron–phonon coupling. This kind of band tail below the bandgap can be described by the so-called Urbach binding energy E_u_, which is the parameter of band tail and can be calculated according to Urbach’s equation [31,32]:

(5) $$1/E_{u}=d(ln\alpha )/d(hv)$$

Figure 3E shows the lnα~eV curve with a steep slope region, the inverse of which gives the Urbach energy E_u_ of GaN on Si. This is like the case of BaTiO_3_ for the determination of E_U_ [33]. It is interesting that all seven GaN films on Si possessed the same E_u_ value, as shown in Figure 3E, indicating that this set of MBE-grown GaN/Si possessed similar film quality even though they had a wide range of film thicknesses which varied between 160 nm and 1500 nm.

### 3.4. Raman (532 nm) and Resonant Raman (325 nm) of Five GaN/Si

**Figure 4:**
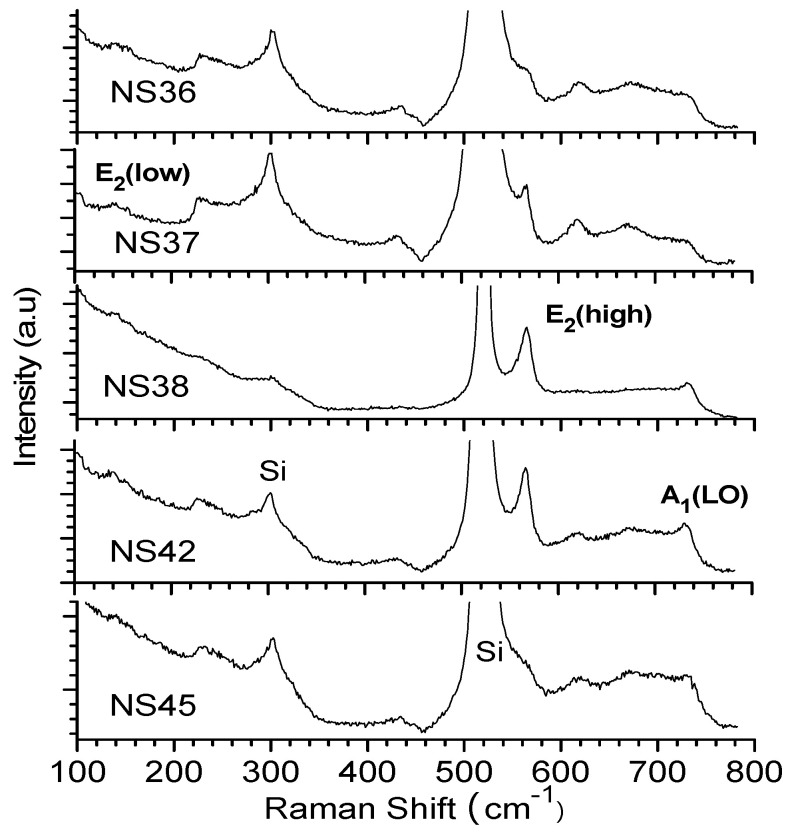
The visible Raman spectra under 532 nm excitation of the five GaN films.

Figure 4 presents the RT visible (532 nm) excitation Raman spectra of five PAMBE-grown GaN films on Si. It shows clearly the E_2_ and A_1_(LO) modes of w-GaN [14,19,29] from the thickest film S3 (NS38, 1522 nm) and the second thickest film S4 (NS42, 579 nm). The E_2_ mode was found to be weaker for S2 (NS37, 204 nm) than for the above two samples, it was also found to be a clear shoulder for S1 (NS36, 182 nm) and appeared as a dim shoulder for S5 (NS45, 315 nm). The A_1_(LO) modes appeared as shoulders for three films of S1, S2, and S5, all of which were thinner than 315 nm.

**Figure 5:**
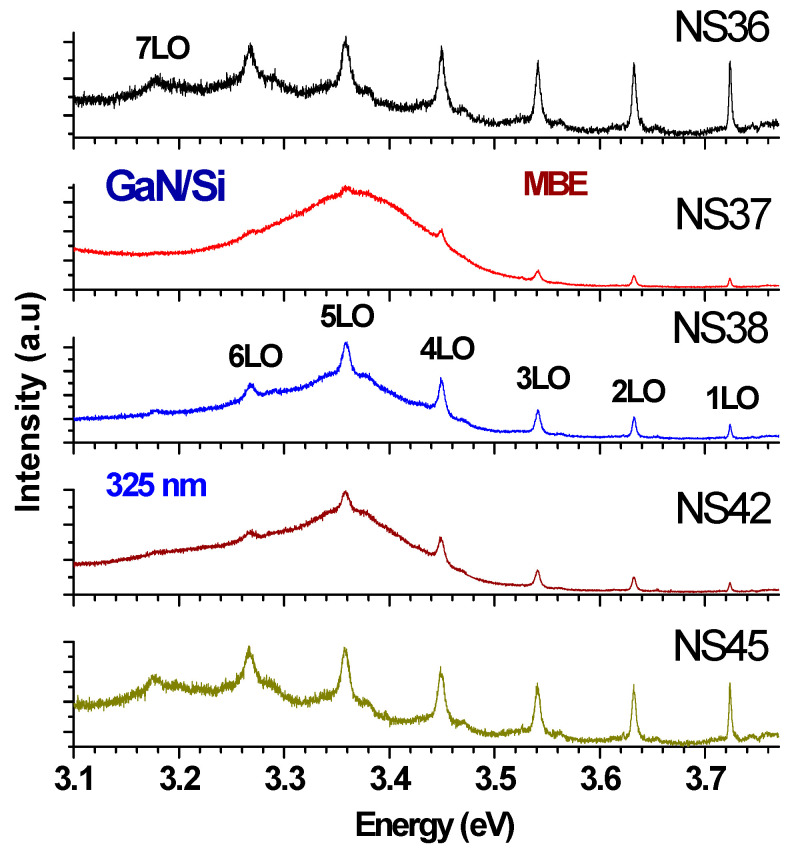
The UV (325 nm) excited PL spectra of five GaN films, with multiple resonant LO phonons.

Figure 5 shows the RT combined PL–Raman spectra under UV (325 nm) excitation measurements for five MBE GaN/Si samples. The three PAMBE GaN/Si samples S2, S3, and S4 (NS37, NS38 and NS42) attained their main PL peak at approximately 3.36 eV. The other two films had their PL band at a similar energy, although it appeared to be weak and overwhelmed by relatively strong LO peaks. Multiple LOs up to 7LO were enhanced because of their outgoing resonance with the E_g_(GaN). The PL peaks of GaN/Si have been shown to be red-shifted compared with the PL peak value of 3.40–3.42 eV from MOCVD-grown GaN/sapphire [29]. This reflects the different hetero-mismatch situation in which the GaN layer on sapphire undergoes a compressive stress due to the sapphire lattice constant being larger than that of w-GaN, while GaN epitaxed on Si has a tensile stress from the reverse situation [34,35].

In addition to the resonance phenomenon, there is another important difference between visible and UV Raman excitations on the light penetration depth, D_p_, which is the inverse of absorption coefficient, α (α = λ/4πk). From Figure 3D, at 532 nm, α~0 and, therefore, D_p_ is very large, and the 532 nm laser light can penetrate through the entire GaN film and Si substrate, leading to Figure 4 for the visible (532 nm) excitation Raman spectra with Si Raman signals overwhelming GaN Raman features. On the other hand, the UV 325 nm had an α near 11,000 cm^−1^ (see the straight blue dash line in Figure 3D), leading to a D_p_ of about 90 nm and detecting only the near surface top layer for the 1500 nm thick S3 (NS38) GaN film. For all the other GaN films thicker than 160 nm (Table 3), the UV 325 nm light was unable to penetrate through the GaN film to reach the Si substrate. Therefore, the UV (325 nm) excitation Raman only showed GaN features without Si signals.

**Figure 6:**
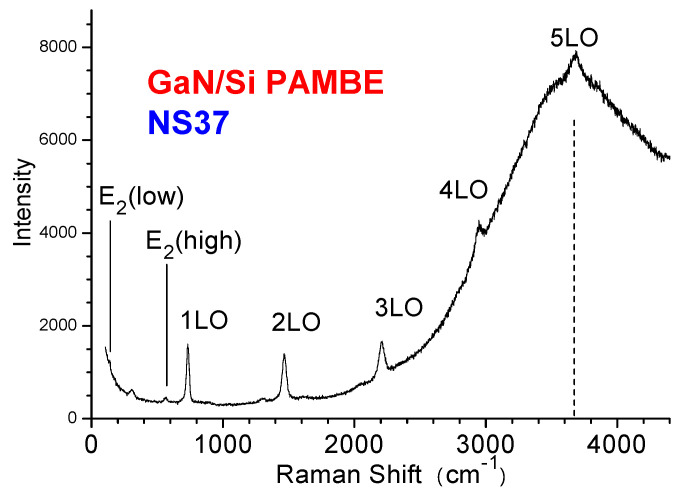
The UV (325 nm) excitation Raman spectrum of GaN/Si sample S2 (NS37), with multiple resonant LO phonons. The GaN E_2_(low) and E_2_(high) phonon modes are indicated. A weak feature at near 300 cm^−1^ is from the Si transverse acoustic (TA) phonon combination. The 5LO is located at the top of the GaN peak at 3670 cm^−1^, i.e., ~3.4 eV.

To demonstrate clearly the multiple LO phonon resonance with the fundamental GaN cross-band gap PL, Figure 6 presents a combined UV–Raman–PL spectrum of an MBE-grown GaN/Si, S2 (NS37), displayed in Raman shift. The broad band centered at approximately 3670 cm^−1^ Raman shifts corresponds to 3.36 eV and represents the emission from the GaN fundamental band edge. GaN Raman E_2_(high) and A_1_(LO) (labeled with 1LO in the figure) modes are observable. Also, because of the resonance with the GaN fundamental band gap of near 3.4 eV at RT, multiple sharp lines appeared superimposed on the top or on the higher energy side of the GaN fundamental recombination band of 3.36 eV, and they are noted as 1LO, 2LO, 3LO, 4LO and 5LO, with the interval of about 732 cm^−1^, i.e., 91 meV, which is the GaN LO phonon energy. This is analogous to the case of GaN on sapphire grown by MOCVD [36].

**Figure 7:**
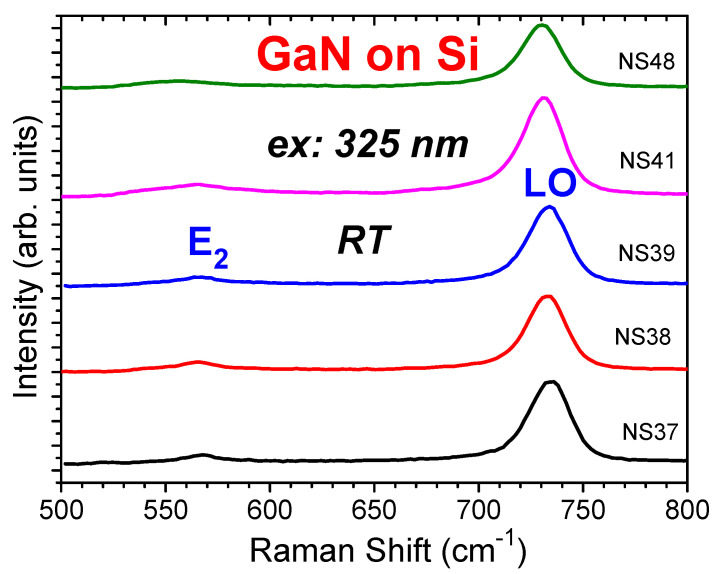
The UV (325 nm) excitation Raman spectra of GaN/Si samples S2 (NS37), S3 (NS38), (NS39), S6 (NS41), and S7 (NS48), respectively, involving GaN phonon modes E_2_(high) and A_1_(LO) or LO.

Figure 7 shows the UV (325 nm) excited Raman spectra of the GaN modes of E_2_, i.e., E_2_(high), and LO, i.e., A_1_(LO), for the samples NS37, NS38, NS39, NS41, and NS48, respectively. Our Raman scattering under an excitation of 325 nm and using a HR T64000 system showed the Raman features of the GaN films only, without the Si Raman 520 cm^−1^ peak, in comparison with the visible (532 nm) excitation Raman spectra in Figure 4. The GaN E_2_(high) phonon modes were very clearly displayed, unlike Figure 4, mixed with the Si 520 cm^−1^ mode.

The GaN E_2_(high) phonon mode can be used to measure the stress [18,37,38]. The GaN with free strain possesses an E_2_(high) phonon frequency of 567.5 cm^−1^ [18]. The Raman frequency difference of the GaN E_2_ mode relative to the strain-free one Δω was used to calculate the layer stress using the formula: σ = Δω/K, where K = −4.3 cm^−1^ GPa^−1^ is the conversion factor of GaN Raman biaxial stress [37,38]. From the Lorentzian fitted E_2_ mode peak values listed in Table 4, S3 (NS38) and S6 (NS41) were found to exhibit tensile stress, while S2 (NS37) and S7 (NS48) were found to exhibit compressive stress.

### 3.5. Raman Spectral Analyses by Spatial Correlation Model of Five GaN/Si

**Figure 8:**
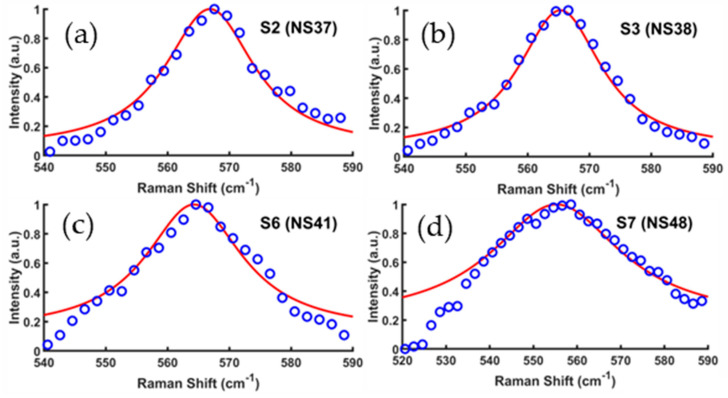
Experimental Raman data and spatial correlation model fits on the E_2_ modes of four typical GaN/Si samples: (**a**) S2 (NS37), (**b**) S3 (NS38), (**c**) S6 (NS41), and (**d**) S7 (NS48).

The E_2_(high) or simply E_2_ modes can also be analyzed by the spatial correlation model (SCM), quantitatively [8,39]. From this model, the frequency (ω)-dependent intensity I(ω) of a first-order Raman spectrum is expressed as:

(6) $$I\left(\omega \right)\propto \int \exp\left(-\frac{q^{2}L^{2}}{4}\right)\frac{d^{3}q}{{\left[\omega -\omega \left(q\right)\right]}^{2}+{\left(\Gamma_{0}/2\right)}^{2}}$$

where q is in units of 2π/a, a is the lattice constant, *L* is the correlation length in units of a, and Γ_0_ is the natural or intrinsic line width. The ω(q), i.e., dispersion relation for optical phonons can be expressed as:

(7) $$\omega^{2}\left(q\right)=A+{\left\{A-B\left[1-\cos\left(\pi q\right)\right]\right\}}^{1/2}\;\mathrm{or}\;\omega \left(q\right)=A-Bq^{2}$$

with *A* and *B* indicating adjustable parameters. Figure 8 shows the fitting results of E_2_ modes for the four MBE-grown GaN/Si samples S2 (NS37), S3 (NS38), S6 (NS41), and S7 (NS48), respectively. Table 4 lists the acquired parameters for the GaN E_2_(high) phonon mode of these GaN/Si samples.

**Figure 9:**
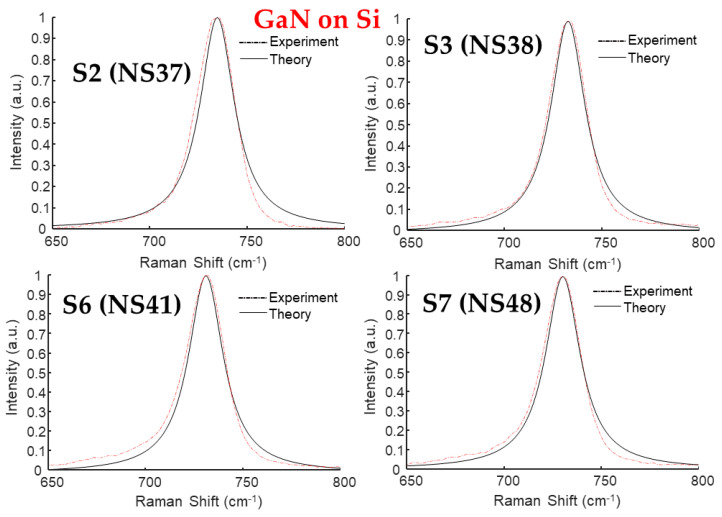
Experimental Raman data and mode fits on the A_1_(LO) modes of four GaN/Si samples.

Raman scattering can offer a non-destructive experimental technique for the determination of the carrier concentration due to doping in semiconductors through the LO phonon and plasma coupling (LOPC). For wide bandgap semiconductors such as SiC, GaN, etc., the Raman LOPC spectral intensity has the following form [36,39,40],

(8) $$I_{LOPC}={\left.\frac{d^{2}S}{d\omega d\Omega }\right|}_{A}=\frac{16\pi hn_{2}}{V_{0}^{2}n_{1}}\frac{\omega_{2}^{4}}{C^{4}}\left(\frac{d\alpha }{dE}\right)\left(n_{\infty }+1\right)AIm\left(-\frac{1}{\varepsilon }\right)\;$$

where *ε* is the dielectric function, *α* is the polarizability, *E* is the macroscopic electric field, *n_ω_* is the Bose–Einstein factor, and *n*_1_ and *n*_2_ are refractive indices at incidence frequency *ω*_1_ and scattering frequency *ω*_2_, respectively. In Equation (8),

(9) $$A=1+2C\frac{\omega_{T}^{2}}{\Delta }\left[\omega_{p}^{2}\gamma \left(\omega_{T}^{2}-\omega^{2}\right)-\omega^{2}\eta \left(\omega^{2}+\gamma^{2}-\omega_{p}^{2}\right)\right]+C^{2}\left(\frac{\omega_{T}^{4}}{\Delta \left(\omega_{L}^{2}-\omega_{T}^{2}\right)}\right)\;\times \left\{\omega_{p}^{2}\left[\gamma \left(\omega_{L}^{2}-\omega_{T}^{2}\right)+\eta \left(\omega_{p}^{2}-2\omega^{2}\right)\;\right]+\omega^{2}\eta \left(\omega^{2}+\gamma^{2}\right)\right\}$$

(10) $$\Delta =\omega_{p}^{2}\gamma \left[{\left(\omega_{T}^{2}-\omega^{2}\right)}^{2}+{\left(\omega \eta \right)}^{2}\right]+\omega^{2}\eta \left(\omega_{L}^{2}-\omega_{T}^{2}\right)\left(\omega^{2}+\gamma^{2}\right)$$

where $\omega_{L}$ is the longitudinal optical (LO) mode frequency, $\omega_{T\;}$ is the transverse optical (TO) mode frequency, *η* is the phonon damping constant, γ is the plasma damping constant, and C is the Faust–Henry coefficient with a value of ~0.35. The dielectric function is expressed as:

(11) $$\varepsilon =\varepsilon_{\infty }\left(1+\frac{\omega_{L}^{2}-\omega_{T}^{2}}{\omega_{T}^{2}-\omega^{2}-i\omega \eta }-\frac{\omega_{p}^{2}}{\omega \left(\omega +i\gamma \right)}\right)$$

(12) $${\;\omega }_{p}^{2}=\frac{4\pi ne^{2}}{\varepsilon_{\infty }m^{*}}$$

where *e* is the electron charge and m* is its effective mass. Figure 9 presents experimental Raman data and mode fits, based on Equations (8)–(12), on the A_1_(LO) modes of four GaN/Si samples.

From Equation (12), the carrier concentration can be expressed as:

(13) $$n=\varepsilon_{\infty }m^{*}\;\omega_{p}^{2}/4\pi e^{2}=M\;{\;\omega }_{p}^{2},$$

where M = $\varepsilon_{\infty }$0.2m_0_/4πe^2^ = 1.1956 × 10^13^ cm^−1^, with $\varepsilon_{\infty }$ = 5.35, m*/m_0_ = 0.2, [39], and with the free electron m_0_ = 9.1 × 10^28^ g and e = 1.602 × 10^−19^ coulombs. Referring to the data of L. Li et al. [40], i.e., an undoped GaN with ω_p_ = 102 cm^−1^ and n = 1.18 × 10^17^ cm^−3^, we obtained M = n/$\omega_{p}^{2}$ = 1.18 × 10^17^/(102)^2^ = 1.134 × 10^13^ (cm^−1^), which matches well with above calculation. We can calculate the carrier concentration n values for four GaN/Si samples, listed in Table 4, which are in the range of 0.5–16 × 10^16^ cm^−3^. The ω_p_ values of our three samples S2, S3, and S7 are better or comparable to the best one of ~2 × 10^16^/cm^−3^ for GaN on Si, reported recently by J. Shen et al. [15].

**Figure 10:**
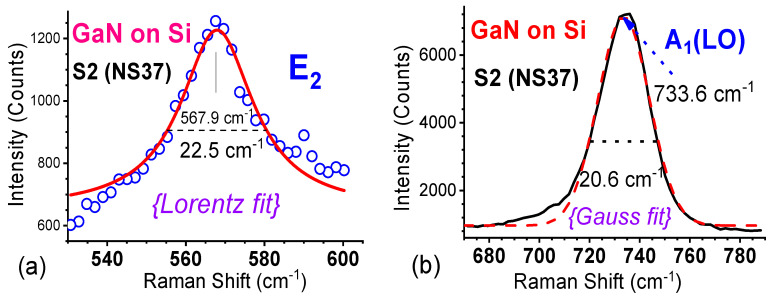
Experimental Raman spectra and fits of (**a**) the E_2_ mode with the Lorentz function and (**b**) the A_1_(LO), i.e., LO mode, with the Gauss function for the GaN/Si sample S2 (NS37).

These Raman modes can also be simply fitted using the Lorentz and Gauss functions. Figure 10 shows experimental Raman data and (a) a Lorentz fit on the E_2_ mode and (b) a Gauss fit on the A_1_(LO), i.e., LO mode, of the GaN/Si sample S2 (NS37). The E_2_(high) phonon had a mode frequency of 567.9 cm^−1^, with FWHM equal to 22.5 cm^−1^. The best fit for A_1_(LO) was the gaussian function, with a peak frequency of 733.6 cm^−1^ and FWHM of 20.6 cm^−1^. These kinds of Lorentz and Gauss fits are suitable for quick characterizations and comparisons on grown materials, especially in the production environment.

### 3.6. Near-Edge X-ray Absorption Fine Structure (NEXAFS) of GaN on Si

Synchrotron radiation (SR) X-ray absorption fine structure (XAFS) technology has been applied to the study of GaN materials [41,42,43,44]. Near-edge X-ray absorption fine structure (NEXAFS) spectroscopy measures the variation of the absorption coefficient versus the incident X-ray photon energy near the absorption edge. It probes the conduction band of crystalline materials, electron dipole transitions, the bonding state, and orientation of adsorbed molecules on surfaces. For K-edge spectra, NEXAFS detects states near the band minimum in the conduction band. The symmetry of the solid and the directions of maximum electron charge densities can be deduced from the NEXAFS spectra and their angular dependence. Here the angular-dependent NEXAFS data from MBE-grown hexagonal GaN are presented and discussed.

**Figure 11:**
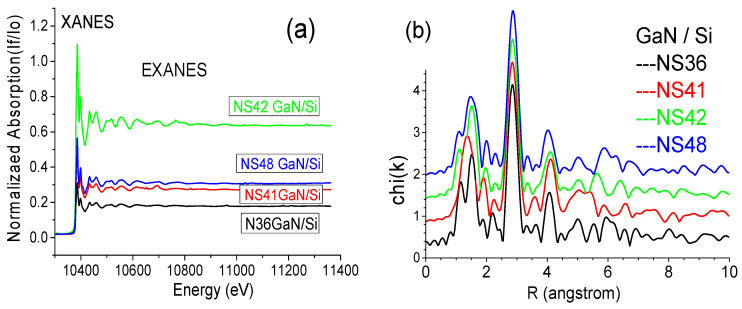
(**a**) EXANES and (**b**) chi(k) vs. R of four GaN/Si samples.

Figure 11a shows the Ga K-edge XAFS spectra of four GaN/Si samples. Two spectral regions are marked: X-ray absorption near edge structure (XANES) near Ga K-edge within about 30 keV, from which information about the local symmetry and various electronic transitions can be obtained [44], and the extended X-ray absorption near edge structure (EXANES), from which information about radial distance and coordination number of the absorbing atom can be deduced [44]. Figure 11b presents their Fourier transformation from k to R space of Ga K-edge EXANES. It is shown that the positions and shapes of the second peak at ~3 Å in the R space are almost the same for the four samples. This means that the Ga–Ga distance in the four GaN films had no difference. However, the Ga–N distance in these samples, ranked from the shortest to the longest, is NS41 < NS48 < NS42 < NS36.

**Figure 12:**
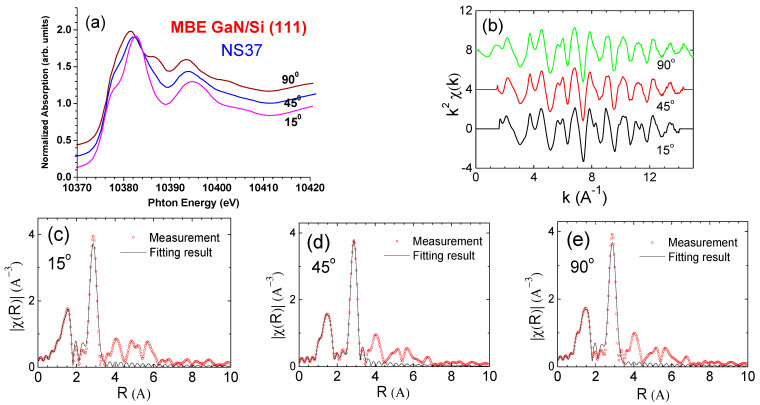
GaN/Si S2 (NS37): (**a**) NEXAFS under three X-ray incident angles, (**b**) k^2^ χ (k) vs. k(1/Å), and (**c**–**e**) EXAFS spectral simulation fits with different X-ray incident angles of 15°, 45°, and 90°, respectively. Red circles represent measurement data and the black lines are fitted spectra, i.e., radial structural functions, derived from the Fourier transformation of the data.

We performed a more penetrative XAFS investigation than that for a typical GaN/Si sample in the case of S2 (NS37). Figure 12a exhibits the angular-dependent NEXAFS spectra from the PA-MBE GaN/Si NS37 sample for three incidences of θ = 15°, 45°, and 90°, all determined from the normal direction of the sample surface. Figure 12b shows the spectral data converted from the energy scale to the *k*-space scale with a *k*^2^-weigthed multiplication. Subsequently, following Figure 12a,b, the Fourier transform of raw data and fitting were done by using the IFEFFIT program [41,44], with results shown in Figure 12c–e. The chi data, i.e., k^2^ χ (k) weighed spectra in Figure 12b, were Fourier transformed to the R-space data. Peaks in spectra in Figure 12c–e indicate reflective waves from the atoms around the absorbed (Ga) atom.

Through the simulation on the EXAFS data, as shown in Figure 2c–e, the nearest Ga–N and the next nearest Ga–Ga (in plan and out plan) bond lengths were obtained. EXAFS data simulations were performed using the IFEFFIT program as follows [41]. First, the EXAFS data at X-ray grazing incidence were fitted to obtain the $R_{GaGa}^{out}$ value for out-of-plane atoms. Subsequently, the EXAFS data at X-ray normal incidence were fitted with a split of Ga–Ga second shell. With the interatomic distance of one of the two subshells fixed to $R_{GaGa}^{out}$ and the other remaining as a fitting parameter, the in-plane Ga–Ga interatomic distance $R_{GaGa}^{in}$ was obtained. It equaled the lattice constant a. Thus, the lattice constant c was obtained by considering the given relation between c and $R_{GaGa}^{out}$. The fitting results are listed in Table 5.

**Figure 13:**
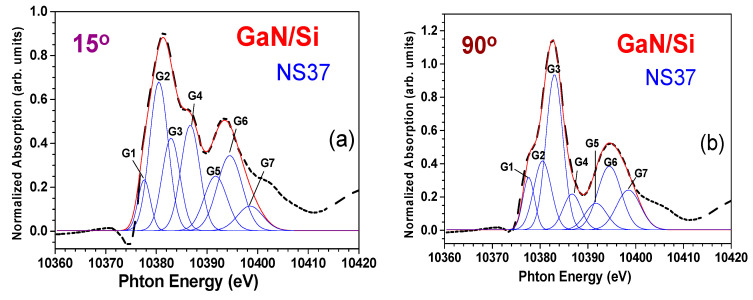
Fittings of NEXAFS spectra of GaN/Si S2 (NS37) sample under two incidence angles of (**a**) θ = 15° and (**b**) θ = 90°. The angle of incidence is determined from the normal direction of the sample surface. The NEXAFS resonances are fitted with gaussian functions, labeled as *G*1–*G*7.

Also, further analyses on the NEXAFS data in Figure 12a revealed more characteristic features of the w-GaN film which are dependent on the incident angle. Specifically, these spectra were properly fitted with gaussian functions for the NEXAFS resonances and indexed as *G*1–*G*7 in Figure 13a,b, which show the angular dependence of the areas under the gaussian functions. The gaussian-function-fitted energy positions, FWHMs, and areas of the NEXAFS resonances from the GaN/Si sample (NS37) are listed in Table 6. Based on these analyses and observations, it was found that the energy positions E and the FWHM of NEXAFS resonances were independent of θ, confirming that this GaN film was purely hexagonal. This conclusion matches with the XRD characterization results.

It is also apparent from Table 6 that the gaussian functions *G*1, *G*3, *G*6, and *G*7 in the NEXAFS resonances represent the transitions from 1s to the final states of *px* and *py* mixing orbitals. Conversely, the gaussian functions *G*2, *G*4, and *G*5 correspond to transitions from 1*s* to other final states of *pz* mixing orbitals. These characteristics from the Ga–K-edge NEXAFS spectrum of GaN obey the hexagonal nature of our GaN sample.

## 4. Conclusions

Thin GaN films were grown on Si using plasma assisted molecular beam epitaxy (PA-MBE), free of cracks and with a thickness of 180–1500 nm. They were investigated by multiple technologies of Nomarski microscopy (NM), high resolution X-ray diffraction (HRXRD), variable angular spectroscopic ellipsometry (VASE), Raman scattering (RS), combined UV micro-Raman–photoluminescence (PL), and synchrotron radiation (SR) near-edge X-ray absorption fine structure (NEXAFS) technique. NM showed no cracks for all GaN films. XRD, Raman, and NEXAFS confirmed their wurtzite crystalline structure with a (0002) orientation. An optimized growth temperature of 700 °C, alongside other corresponding parameters, were obtained to achieve high-quality GaN thin films on Si.

VASE and simulations precisely determined the thickness and band gaps of GaN films and deduced the Urbach energy. The UV (325 nm) excited Raman measurements exhibited the GaN E_2_(low) and E_2_(high) phonon modes clearly without Raman features from the Si substrate, overcoming the difficulties from visible (532 nm) Raman measurements, and revealed multiple LO phonons due to the outgoing resonance effect. This GaN PL E_o_ peak was located at 3.36 eV for GaN/Si, red shifted compared to 3.40–3.42 eV from MOCVD-grown GaN/sapphire. This reflects the different hetero-mismatch with compressive stress of GaN on sapphire due to the sapphire lattice constant being larger than that of w-GaN, while, with respect to tensile stress for GaN/Si, the opposite was true. The spatial correlation model (SCM) was applied to quantitatively analyze the E_2_ modes for the GaN/Si samples. The plasma frequency ω_p_ was obtained and the carrier concentrations were calculated for GaN/Si samples with excellent values.

EXANES indicated that the Ga–Ga distance was the same across the four GaN films, although the Ga–N distances were different. Angular-dependent NEXAFS revealed the significant anisotropy of the conduction band of w-GaN. NEXAFS resonance fits showed the area dependent on the incidence angle, while the energy positions and FWHMs were independent of the angle; it also revealed the transitions from 1s to two types of final states.

All of the above results are significant and attractive for further development of high-quality nitride materials and related Si devices.

## Figures and Tables

**Table 1:** PA-MBE growth and sample information.

Sample No.	S1	S2	S3	S4	S5	S6	S7
Initial run no.	NS36	NS37	NS38	NS42	NS45	NS41	NS48
Growth temp. (^°^C)	650	700	700	700	650	650	750
Growth time (min.)	120	120	600	120	120	120	120
Ga flux (×10^−7^ torr)	2.1	2.0	2.1	1.9		2.5	2.5
Thickness (nm)–from SE	162	175	1507	547	298	262	248
Overview of NM	Fair	Best	Poor	Worst	Good	Worst	Good
PL GaN peak (RT)	Weakest	Strongest	Strong	Strong	Weak		
Raman GaN E_2_ peak	Clearly	As a shoulder	Strong	Strong, narrowest	As a dim shoulder		
Raman GaN A_1_(LO) peak	As a shoulder	As a shoulder			As a shoulder		

**Table 2:** Values of (0002) 2θ peak/FWHM and calculated results of crystallite size, lattice strain, and dislocation density of five GaN films on Si.

Sample No.	S1	S2	S3	S4	S5
Initial run no.	NS36	NS37	NS38	NS42	NS45
Peak 2θ (0002) (°)	34.52	34.49	34.50	34.51	34.48
FWHM 2θ (0002) (°)	0.254	0.216	0.205	0.219	0.356
β: (π/180, Rad)	0.004433	0.00377	0.003578	0.003822	0.006213
β cosθ	0.004234	0.00360	0.00342	0.00365	0.005934
Crystallite size D (nm)	32.76	38.53	40.56	38.0	23.37
Lattice strain (×10^−3^)	1.058	0.90	0.854	0.913	1.48
Dislocation density (×10^10^) (cm^−2^)	9.32	6.74	6.08	6.93	18.31

**Table 3:** SE fitting results of seven GaN films on Si.

Sample No.	S1	S2	S3	S4	S5	S6	S7
Initial run no.	NS36	NS37	NS38	NS42	NS45	NS41	NS48
Surface roughness (nm)	3.3	2.7	8.3	4.9	7.1	3.3	10.5
GaN thickness (nm)	162	175	1507	547	298	262	248
AlN buffer thickness (nm)	20.0	20.0	20.0	20.0	20.0	20.0	20.0
GaN band gap by SE (nm)	362.95	362.80	362.64	362.64	362.79	362.80	363.13
GaN band gap by SE (eV)	3.416	3.418	3.419	3.419	3.418	3.418	3.415
AlN buffer gap by SE (nm)	197.64	197.32	197.48	197.63	197.48	197.32	
AlN buffer gap by SE (eV)	6.274	6.284	6.279	6.274	6.279	6.284	
E_u_ by SE (meV)	18.3	18.3	18.3	18.3	18.3	18.3	18.3

**Table 4:** A list of Raman E_2_ and A_1_(LO) mode fitting results of four GaN/Si samples.

Sample No.	S2	S3	S6	S7
Initial run no.	NS37	NS38	NS41	NS48
E_2_ (cm^−1^)-Lorentz fit	567.9	565.9	568.5	559.2
E_2_ FWHM (cm^−1^)-Lorentz fit	22.5	40.0	58.3	66.9
SCM fit A (cm^−1^)	568.3	566.5	565	556.5
SCM fit B (cm^−1^)	109.5	100	101	100
SCM fit L (Å)	21	22	29	22
SCM fit Γ_0_ (cm^−1^)	17	16	19	36
ω_p_ (cm^−1^) from LO fit	120	90	50	20
Carrier concentration n (×10^16^ cm^−3^)	16.3	9.2	2.8	0.45

**Table 5:** Simulation results of the Fourier transform Ga K-edge EXAFS spectra.

Angel	R^Ga-N^ (Å)	R^Ga-Ga^_in-plan_ (Å)	R^Ga-Ga^_out-plan_ (Å)
15°	1.936		3.193
45°	1.984	3.176	3.294
90°	1.920	3.195	

**Table 6:** NEXAFS resonances: gaussian-function-fitted values of the energy position, FWHM, and area for the GaN/Si sample (NS37).

R_esonance_	G1		G2		G3		G4		G5		G6		G7	
I_ncident angle_	15°	90°	15°	90°	15°	90°	15°	90°	15°	90°	15°	90°	15°	90°
E (ev)	10,377.6		10,380.5		10,383.5		10,386.7		10,391.7		10,394.5		10,398.5	
FWHM (ev)	2.4		3.65		3.5		3.7		4.5		4.9		5	
Area (arb. Units)	0.7	0.95	3.1	1.9	1.85	4.1	2.23	1	1.4	0.9	2.1	2.35	0.7	1.5
T_ransition_	1s => p_xy_		1s => p_z_		1s => p_xy_		1s => p_z_		1s => p_z_		1s => p_xy_		1s => p_xy_	

## Data Availability

The data that support the findings of this study are available from the leading author, Z.C.F., upon reasonable request.
